# Late Onset Remnant Gastric Cancer with Afferent Loop Syndrome 47 Years after Billroth II Surgery

**DOI:** 10.1155/2015/730897

**Published:** 2015-05-10

**Authors:** Memduh şahin, Bahattin Ozlu, Kivilcim Eren Erdogan, Tahsin Colak

**Affiliations:** ^1^Gastroenterology Department, Endoscopy Unit, Mersin State Hospital, Nusratiye Mahallesi, 33050 Mersin, Turkey; ^2^Gastrointestinal Surgery Department, Mersin State Hospital, 33050 Mersin, Turkey; ^3^Pathology Department, Mersin State Hospital, 33050 Mersin, Turkey; ^4^General Surgery Department, Mersin University, 33050 Mersin, Turkey

## Abstract

Remnant gastric cancer is a rare clinical entity. Herein we describe a patient with remnant gastric cancer that presented with afferent loop syndrome 47 years after Billroth II surgery. Symptoms of serious bilious vomiting were an indication to perform early endoscopic diagnosis, followed by complete gastric resection. In particular, patients that have undergone surgery due to benign indications should be examined endoscopically, even a long time after initial surgery.

## 1. Introduction 

Remnant gastric cancer (RGC) is a rare clinical entity that can occur following distal gastrectomy, especially along the suture line or in the anastomotic region [1]. The occurrence of RGC in gastric cancer patients following distal gastrectomy was reported to be approximately 1%-2% in a Japanese study [2]. RGC has similar metastatic characteristics and surgical treatment procedures as gastric cancer [1]. RGC is generally diagnosed at an advanced stage, with a low chance of cure, high rate of lymph node metastasis, and poor prognosis [3]. Recent advances in diagnosis and treatment have increased the rate of detection of RGC following distal gastrectomy [4]. The standard surgery for RGC is complete gastric resection and lymph node dissection. It was reported that as time after gastric surgery increases, the risk of gastric adenocarcinoma increases. Approximately 70%–75% of gastric remnant carcinomas are resectable and 60%–70% are removed for complete cure. Although adjuvant chemotherapy and radiotherapy have been suggested, their efficacy remains unclear [5].

Afferent loop obstruction is a rare entity following Billroth II reconstruction and subtotal gastrectomy [6]. The onset can present as acute or late and can be accompanied by peritonitis and/or perforation, which can result in death. The incidence of afferent loop obstruction following Billroth II surgery is 0.3%–1%. Herein we describe a patient with RGC and afferent loop syndrome that presented 47 years after Billroth II surgery.

## 2. Case

A 70-year-old Caucasian male presented to the Mersin State Hospital, Department of Gastroenterology, Mersin, Turkey, with vomiting and abdominal pain. Medical history showed weight loss up to 10 kg during the previous 3 months due to frequent bilious vomiting and colic abdominal pain, which restricted oral intake. The patient had undergone gastric surgery (distal gastrectomy and Billroth II reconstruction) 47 years earlier (1967) because of a peptic ulcer. The patient had a negative history of constipation and diarrhea. The patient had previously presented to numerous other medical centers with the same symptoms and had been unsuccessfully treated with proton pump inhibitors (PPIs) and antiacid agents. Physical examination showed a pale tongue and epigastric tenderness. He also had a cachectic body composition. Laboratory findings showed moderate anemia (hemoglobin: 9.89 g dL^−1^) and low serum albumin (3.31 g dL^−1^) and sodium (133 g dL^−1^), indicative of poor nutrition. Other biochemical parameters were in the normal range.

**Figure 1:**
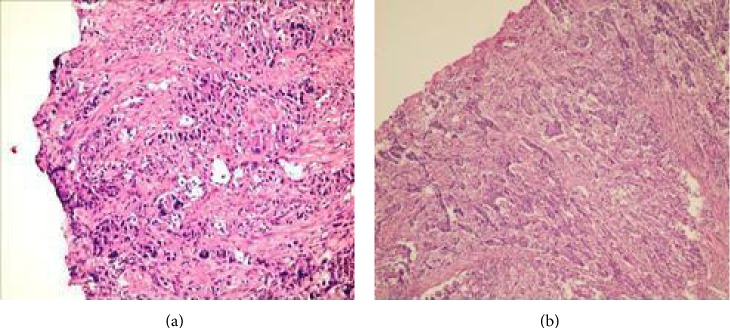
(a) Adenocarcinoma diagnosed via endoscopic biopsy (H&E, ×100). (b) Adenocarcinoma extending from the mucosa to the serous surface (H&E, ×100).

**Figure 2:**
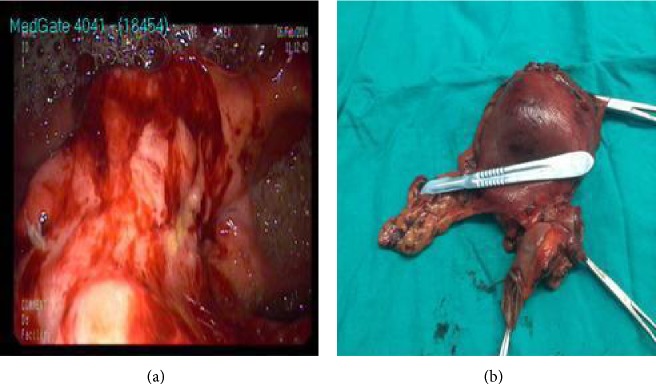
(a) Gastric mass ulceration on the anastomosis (via endoscopy). (b) The resected gastric material with mass infiltration of the afferent loop.

Abdominal and thoracic CT findings were normal liver and spleen parenchyma without any intra-abdominal fluid collection and an emphysematous lung disorder. Upper endoscopy showed an approximately 5 cm diameter ulcerovegetant mass with irregular borders on the gastrojejunostomy anastomosis, which was obstructing a major portion of the intestinal lumen, not allowing passage of the afferent loop. Biopsy of the mass showed a poorly differentiated adenocarcinoma (Figure 1(a)). The fundic and esophageal junctions were normal (Figure 2(a)).

Following preoperative preparation, the patient was scheduled for open surgery. A midline incision was performed and the explorative finding was an approximately 5 cm palpable, irregular severe obstruction and a dilated preobstructive afferent loop; however, there were no signs of locoregional or distal metastasis (Figure 2(b)). The neoplastic mass began on the gastrojejunal anastomosis and extended through the afferent loop. The total remnant stomach and affected intestines were resected with safe clear margins and omentectomy was also performed. Reconstruction was accomplished using Roux-en-Y esophagojejunostomy. Splenectomy was performed at the end of the surgery because of splenic injury caused by severe bleeding.

Histopathologic examination of the specimen showed serous adenocarcinoma with perineural infiltration. All surgical margins were intact and immunohistochemical examination was cerbB2 negative (Figure 1(b)). An anastomotic leak developed on postsurgical d 3. Fortunately, the leak was successfully managed with supportive therapy and enteral feeding via nasojejunal tube. The leak stopped on postsurgical d 20 and regular oral intake commenced. The patient was discharged on postsurgical d 30, at which time his final stage was T3 NO MO, requiring subsequent adjuvant chemotherapy; therefore, he was referred to the oncology department.

## 3. Discussion 

The lifetime risk of RGC following gastric resection due to peptic ulceration is ≤10% [7]. Environmental factors affecting the remnant gastric mucosa after primary distal gastric resection are thought to be the cause of malignant transformation. The most prominent environmental factors are duodenogastric reflux of bile and pancreatic juice [1]. The presentation of afferent loop syndrome due to RGC is an extremely rare event. Generally, RGC with afferent loop syndrome has a poor prognosis. Kawaoka et al. [8] reported a case of RGC with symptoms of afferent loop syndrome. Left upper exenteration was performed because of transverse colon, pancreas, and lateral segment of the liver and left renal hilus involvement. In contrast, the presented case had stage T3 N0 M0 gastric cancer that was treated with total gastrectomy. Splenectomy was also performed in the presented case because of a limited bleeding episode. There were not any signs of local metastasis in the presented case's postoperative gastric, spleen, or lymph node biopsy samples.

Contrary to earlier reports, Komatsu et al. [9] reported that >50% of RGC cases were diagnosed in the early stage (T1/2), with the potential for complete resectability. They also reported that RGC was diagnosed at a mean of 12 years (range: 2–22 years) following gastrectomy performed to treat malign conditions versus a mean of 30 years (range: 4–51 years) after gastrectomy performed because of benign conditions. In addition, they noted that RGC occurred significantly more frequently after Billroth I surgery than after Billroth II surgery. Their findings indicate that endoscopic follow-up of distal gastrectomy should be reevaluated for early detection of RGC. So our report demonstrates that clinical signs associated with afferent loop syndrome could be alarm symptoms and an indication for early RGC detection. Sudden weight loss and bilious vomiting should be considered a strong indication for upper endoscopy. Based on those findings, the presented case exhibited the typical presentation of cancer cell infiltration of the anastomosis that was performed 47 years earlier. Maybe the bile involvement caused an insidious malignant transformation during this long period.

Ojima et al. [10] compared benign and malign indications for gastric surgery and the mean postoperative time to RGC in cases that developed RGC. The mean time from gastric surgery to RGC was shorter in cases with malign indications for surgery than in those with benign indications (9 ± 10 years versus 22 ± 9 years, *P* < 0.001). RGC in most of the patients with malign indications was located in the nonanastomotic region of the stomach. RGC volume in the patients with benign indications for surgery was larger than in those with malign indications. Lastly, TNM stage differed significantly between the 2 groups (TNM stage [I/II/III/IV] in patients with malign indications for surgery: *n* = 13/0/0/8; TNM stage [I/II/III/IV] in patients with benign indications for surgery: *n* = 6/4/3/4). Based on their findings, they recommended annual follow-up for all gastric surgery patients. Our case was operated for a benign reason but then developed voluminous malignant infiltration located in the anastomosis and this made us think that evaluation of afferent loop syndrome should be taken into consideration for early endoscopic intervention. Other rare malignancies that have been reported to occur following gastric surgery include sarcomatoid carcinoma [11] and squamous carcinoma [12]; in both cases, they were located in the gastric cardiac region, unlike in the presented case.

## 4. Conclusion 

Based on the presented case, RGC is a malignancy that can be diagnosed and surgically treated following the observation of early diagnostic symptoms, including sudden bilious vomiting and afferent loop syndrome. The presented patient was diagnosed as RGC 47 years after gastric surgery. In particular, patients that have undergone gastric surgery for benign indications should be endoscopically examined even though it has been a long time since the first surgical procedure was performed. Although the incidence of gastric surgery for benign conditions is decreasing, as RGC in benign cases occurs in the late postoperative period, lifetime annual endoscopy is recommended for early diagnosis. Sudden weight loss and severe bilious vomiting 47 years following gastric surgery for benign conditions are alarm symptoms or warning signs and an indication for early endoscopic monitoring.

## Abbreviations

RGC: Remnant gastric cancer.

## References

[1] Firat O., Guler A., Sozbilen M., Ersin S., Kaplan H. (2009). Gastric remnant cancer: an old problem with novel concerns. *Langenbeck's Archives of Surgery*.

[2] Ohashi M., Katai H., Fukagawa T., Gotoda T., Sano T., Sasako M. (2007). Cancer of the gastric stump following distal gastrectomy for cancer. *British Journal of Surgery*.

[3] Tanigawa N., Nomura E., Lee S.-W. (2010). Current state of gastric stump carcinoma in Japan: based on the results of a nationwide survey. *World Journal of Surgery*.

[4] Nakayoshi T., Tajiri H., Matsuda K., Kaise M., Ikegami M., Sasaki H. (2004). Magnifying endoscopy combined with narrow band imaging system for early gastric cancer: correlation of vascular pattern with histopathology. *Endoscopy*.

[5] Holzheimer R. G., Mannick J. A. (2001). *Surgical Treatment: Evidence-Based and Problem-Oriented*.

[6] Wada N., Seki M., Saikawa Y. (2000). Jejunal limb obstruction caused by a cholesterol stone 15 years after a total gastrectomy and 20 years after a cholecystectomy: report of a case. *Surgery Today*.

[7] Kondo K. (2002). Duodenogastric reflux and gastric stump carcinoma. *Gastric Cancer*.

[8] Kawaoka T., Kuwahara T., Kaneko T., Harada T., Hiraki S., Fukuda S. (2013). A case of remnant gastric cancer with afferent loop syndrome treated by left upper exenteration. *Gan To kagaku Ryoho*.

[9] Komatsu S., Ichikawa D., Okamoto K. (2012). Progression of remnant gastric cancer is associated with duration of follow-up following distal gastrectomy. *World Journal of Gastroenterology*.

[10] Ojima T., Iwahashi M., Nakamori M. (2010). Clinicopathological characteristics of remnant gastric cancer after a distal gastrectomy. *Journal of Gastrointestinal Surgery*.

[11] Sato A., Oki E., Kohso H. (2013). Sarcomatoid carcinoma of the remnant stomach: report of a case. *Surgery Today*.

[12] Tokuhara K., Nakano T., Inoue K., Nakane Y., Kwon A.-H. (2012). Primary squamous cell carcinoma in the gastric remnant. *Surgery Today*.

